# Supplementary material for: Non-Alcoholic Fatty Liver Disease Is Associated with Higher Metabolic Expenditure in Overweight and Obese Subjects: A Case-Control Study
Source: Nutrients. 2019 Aug 7;11(8):1830. doi: 10.3390/nu11081830 (PMC6723627; doi:10.3390/nu11081830)
Supplement: Supplementary file 1 [file nutrients-11-01830-s001.pdf]

**Supplementary Table S1.** Grading used for liver echography.

| Parameter | − | + | ++ |
|---|---|---|---|
| **Appearance of the liver echotexture** | No difference between kidney and liver parenchyma | Mild discrepancy between liver and kidney parenchyma | Large discrepancy between liver and kidney parenchyma |
| **Hepatic echo penetration** | Liver parenchyma visible up to the diaphragm | Diaphragm not visible or presence of opacities | Diaphragm not visible and presence of opacities |
| **Clarity of the hepatic blood vessels** | Hepatic blood vessels clearly visible | Loss of echoes of hepatic blood vessels | Hepatic blood vessels not clearly visible |
